# A comprehensive analysis of the phylogenetic signal in ramp sequences in 211 vertebrates

**DOI:** 10.1038/s41598-020-78803-3

**Published:** 2021-01-12

**Authors:** Lauren M. McKinnon, Justin B. Miller, Michael F. Whiting, John S. K. Kauwe, Perry G. Ridge

**Affiliations:** 1 Department of Biology, Brigham Young University, Provo, UT 84602 USA; 2 Monte L. Bean Museum, Brigham Young University, Provo, UT 84602 USA

**Keywords:** Phylogenetics, Evolutionary genetics, Evolutionary biology, Genomics

## Abstract

Ramp sequences increase translational speed and accuracy when rare, slowly-translated codons are found at the beginnings of genes. Here, the results of the first analysis of ramp sequences in a phylogenetic construct are presented. Ramp sequences were compared from 247 vertebrates (114 Mammalian and 133 non-mammalian), where the presence and absence of ramp sequences was analyzed as a binary character in a parsimony and maximum likelihood framework. Additionally, ramp sequences were mapped to the Open Tree of Life synthetic tree to determine the number of parallelisms and reversals that occurred, and those results were compared to random permutations. Parsimony and maximum likelihood analyses of the presence and absence of ramp sequences recovered phylogenies that are highly congruent with established phylogenies. Additionally, 81% of vertebrate mammalian ramps and 81.2% of other vertebrate ramps had less parallelisms and reversals than the mean from 1000 randomly permuted trees. A chi-square analysis of completely orthologous ramp sequences resulted in a p-value < 0.001 as compared to random chance. Ramp sequences recover comparable phylogenies as other phylogenomic methods. Although not all ramp sequences appear to have a phylogenetic signal, more ramp sequences track speciation than expected by random chance. Therefore, ramp sequences may be used in conjunction with other phylogenomic approaches if many orthologs are taken into account. However, phylogenomic methods utilizing few orthologs should be cautious in incorporating ramp sequences because individual ramp sequences may provide conflicting signals.

## Introduction

The central dogma of biology states that DNA is transcribed into RNA, which is subsequently translated in sets of three consecutive nucleotides, called codons^1^. Since there are 61 possible codons (and three stop codons) which encode only 20 amino acids, there is redundancy in the genetic code. Although synonymous codons encode the same amino acid, recent research has shown that translational efficiency differs between synonymous codons^2–4^. These differences cause a change in translational speed, which affects gene expression^5,6^. Ramp sequences consist of 30–50 infrequent or slowly-translated codons (i.e., codons translated by a relatively small proportion of tRNA molecules from the tRNA pool) at the 5′ end of many genes^7–9^. These sequences serve as a means to regulate gene expression by evenly spacing ribosomes along the mRNA transcript to reduce downstream ribosomal collisions^8^ and reduce mRNA secondary structure at translation initiation^10^. Additionally, variation of translation rate plays a regulatory function in protein folding, indicating that the slower translation rate in the ramp sequence may serve to direct the nascent protein in the correct folding pathway^11–13^.

We recently developed an algorithm, ExtRamp^9^, to identify the presence of a ramp sequence in a gene sequence. Previously, ramp sequences were known and characterized in only a few model species. ExtRamp identifies ramp sequences by calculating the relative codon efficiency of each codon and then estimating ribosomal speed at each location in the gene by computing the average codon efficiency within the ribosomal window. If an outlier portion is present at the beginning of the gene, it is considered a ramp sequence. Using this algorithm, about 10% of genes in most species across most domains of life were shown to contain ramp sequences^9^. Given the widespread presence of ramp sequences in most domains of life and their role in regulating translation, here we investigate the hypothesis that the presence or absence of a ramp sequence in a gene may be used as a morphological genomic character that can be used to recover a phylogenetic signal.

Phylogenies are essential to understanding the biological world and allow biologists to analyze similarities and differences between closely related species^14^. They also provide an evolutionary context to better understand biological processes and patterns. Our knowledge of phylogenetic relationships increases in accuracy as more phylogenetically informative data are incorporated into tree reconstruction. In order to analyze the ever-increasing amounts of phylogenetic data, many methods and frameworks of phylogenetic inference have been developed, each of which seeks to determine species relationships according to a set of assumptions of evolutionary processes. Maximum likelihood is a statistical method that incorporates a model of evolution (e.g., transition and transversion frequencies, nucleotide frequencies, evolutionary rates, etc.) to take into account the likelihood of evolutionary events^15^. Parsimony seeks to maximize homology in phylogenies by minimizing ad hoc hypotheses of homoplasy^16^. Although the algorithms differ, each phylogenomic method requires the underlying data to contain an accurate phylogenetic signal. Since nearly all characters, including morphological and molecular, contain some amount of homoplasy^17^, incorporating additional phylogenetically informative characters into each algorithm increases the overall accuracy of the recovered phylogeny. Therefore, we analyzed the potential of ramp sequences to be used as a novel phylogenetic character state in order to determine the extent to which ramp sequences can be incorporated in phylogenomic analyses. Additionally, we investigate the possibility that ramp sequences display a different phylogenetic signal than other portions of the sequence. The potential for using ramp sequences in future phylogenomic studies is then evaluated.

## Methods

### Data collection and processing

Reference genomes were downloaded along with their corresponding General Feature Format (GFF3) files from the National Center for Biotechnology Information (NCBI) database^18–21^ in August 2018 using the NCBI FTP site: ftp://ftp.ncbi.nlm.nih.gov/genomes/refseq/. We used the most recent reference assembly version for each of 247 vertebrate species (see Supplementary Notes S1 and S2 for list of species used in this study). The mammalian taxonomic group was analyzed (114 mammalian species), as well as their non-mammalian vertebrate outgroup (133 non-mammalian species). Our analyses include only vertebrate species because insufficient orthologous ramp sequences were identified in other taxonomic groups. Of archaea, bacteria, fungi, invertebrates, mammalian vertebrates, other vertebrates, plants, protozoa, and viruses, only vertebrates passed our filtering criteria to ensure orthologs contained ramp sequences in at least 5% of the available species and did not contain ramp sequences in at least 5% of the available species. At least 5% of all annotated orthologs needed to pass those filtering criteria for a taxonomic group to be included in our analyses.

We then assessed the congruence of the phylogenetic signal of ramp sequences within mammalian species and their vertebrate outgroup. All coding sequences (CDS) data were extracted from the reference genomes using a GFF3 parser included in JustOrthologs^22^. Any sequences with annotated exceptions, such as translational exceptions, unclassified transcription discrepancies, and suspected errors, were removed from the dataset. Our analyses included all NCBI gene annotations. NCBI gene annotations are calculated by NCBI's Eukaryotic Genome Annotation pipeline for the NCBI Gene dataset. They use a combination of protein sequence similarity and local synteny information to establish orthology. A manual curator may additionally assign orthologous gene relationships. The NCBI database includes 34,202 orthologs for Mammalia and 41,337 orthologs for non-mammalian vertebrates.

### Identifying ramp sequences

**Figure 1:**
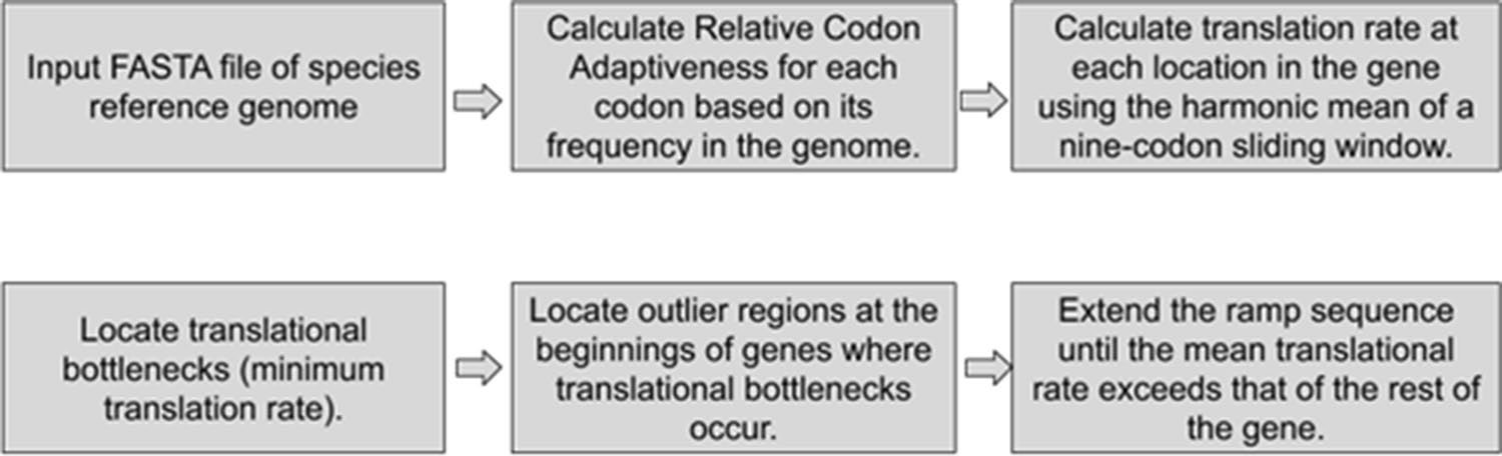
Identifying Ramp Sequences Using ExtRamp. Flowchart for finding ramp sequences using ExtRamp.

Ramp sequences were identified using ExtRamp (Fig. 1). The relative codon adaptiveness was calculated for each codon by using its frequency in the genome. The translation rate at each codon in the gene was then estimated using the mean translational efficiency of a window of codons. A nine-codon sliding window was used to approximate the span of a ribosome, as recommended in the ExtRamp documentation^9^. Ramp sequences were identified when low outlier regions of codon translational efficiency (i.e., a translational bottlenecks) occurred at the beginning of gene sequences. ExtRamp was run on each species FASTA file (.fasta) containing all genes using the options to output the ramp sequence and the portion after the ramp sequence, as described in the ExtRamp README file (https://github.com/ridgelab/ExtRamp) The exact command used is included in Supplementary Note S3.

### Recovering phylogenies using the presence and absence of ramps

The presence or absence of a ramp sequence in each annotated ortholog was encoded in a binary matrix. If a ramp sequence was present in an ortholog, it was encoded in the matrix as a '1’, and if it was absent, it was encoded as a '0’. Species that did not contain the ortholog were assigned a '?' for a missing value, similar to other methods that have found phylogenetic signals in codon usage biases^23–25^. The effect of missing data was limited by applying an additional filter to the data. An orthologous gene was included in the analyses only if a ramp sequence in that gene was found in at least 5% of the species. Additionally, all species were required to contain ortholog annotations for at least 5% of the orthologs passing that initial filter. After applying this filter, mammalian species had a mean of 16.31% ± 7.81% missing data, and non-mammalian vertebrates had a mean of 28.50% ± 13.11% missing data.

Parsimony phylogenetic trees were recovered using Tree Analysis using New Technology (TNT)^26^. The most parsimonious trees were found by saving multiple trees using tree bisection reconnection (tbr) branch swapping^27^. Maximum likelihood trees were recovered using IQTREE^28^.

### Retrieving reference phylogenies

In order to determine the congruence of the phylogenetic signal of ramp sequences, each of the recovered phylogenies (i.e., parsimony and maximum likelihood trees) were compared to the synthetic phylogeny from the Open Tree of Life (OTL)^29^. Although this phylogeny cannot be considered the "true" tree, it is created from a conglomeration of many phylogenetic studies, and provides a useful resource for benchmarking ramp sequences as a new character state. The synthetic phylogeny was retrieved from the OTL using a previously-published parser, getOTLtree.py^30^, that references the OTL application programming interface (API) to obtain OTL taxonomy identifiers for each species and retrieves the phylogeny from the OTL database. The exact command is included in Supplementary Note S4.

### Comparisons with the OTL synthetic tree

The accuracy of recovered phylogenies based on ramp sequence presence or absence were assessed by comparing each tree to the OTL synthetic phylogeny. The difference was quantified using branch percent comparisons, as implemented by the Environment for Tree Exploration toolkit ete3 compare module^31,32^. This metric computes the percentage of branch similarity between two trees, where a high percentage corresponds to more similar trees. This metric was selected because of its ability to compare large trees, including unrooted trees and trees with polytomies. The baseline performance of the ete3 branch percent identity metric was determined by comparing 1000 random permutations of the mammalian and other vertebrate topologies to the OTL.

### Scoring ramp sequences

Using the binary matrix of ramp sequences within each ortholog, the extent to which ramp sequences are homoplasious was quantified by mapping each ramp sequence to the OTL. For each ramp sequence, the species were divided into two partitions based on presence or absence of the ramp sequence. Since autapomorphies do not provide phylogenetic information, an orthologous ramp sequence was required to be present in at least two species and absent in at least two species, assuming a fully-resolved tree. For each ramp sequence, the number of parallelisms and reversals that occurred was quantified. Parallelisms occur when a character arises independently multiple times due to convergent evolution. Reversals occur when a derived character is lost or when the character reverts back to its ancestral state. A ramp sequence was determined to be orthologous if it correctly separated species according to their relationships reported in the OTL, and if the total number of gain/loss events equaled one, as previously computed for other codon usage biases^23,24^. The number of origin and loss events was then used to calculate the retention index for each ramp sequence^33^, where a retention index of zero represents a fully homoplasious character, and a retention index of one represents a character in which none of the states are homoplasious.

### Statistical calculations using random permutation test

Random permutations were performed in order to determine the extent to which the observed mean retention index of ramp sequences compares to random chance. Permutation tests (also called randomization tests) are non-parametric statistical tests that determine statistical significance by randomly rearranging the labels of a dataset^34^. The taxa in the OTL were shuffled 1000 times to generate random trees. The tree topology of the OTL was maintained to prevent any biases due to tree topology. The retention indices of the ramp sequences were calculated for each random tree to create a null distribution of retention indices due to random chance. The actual mean retention index of the ramp was compared to this distribution and an empirical p-value was calculated as the proportion of permutated retention indices less than or equal to the observed retention index from the OTL.

### Statistical calculation of completely orthologous ramps

A ramp sequence was considered orthologous if all species that either have or do not have the ramp sequence form a monophyletic group. For each orthologous ramp sequence, the probability that it would form a monophyletic group in agreement with the OTL topology due to random chance was calculated. The species were divided into two groups: species with ramp sequences, and species without ramp sequences. The conditional probability was then calculated that a group of species would randomly divide into a monophyletic group concordant to the OTL using the method previously described in Miller, et al.^23^, which describes how *(t)* total species with *(s)* number of species in the smaller of the two groups (i.e., species with ramps or species without ramps for a given gene) will track a proposed phylogeny using Eq. (1).

1 $$\frac{{\prod }_{i=1}^{s-1}i}{{\prod }_{j=t-s+1}^{t-1}j}$$

For example, if three species contain a ramp sequence in an orthologous gene and there are seven total species, then the probability that the three species containing a ramp sequence in the orthologous gene would form a monophyletic group in agreement with the OTL topology by random chance is as follows:

$$P= \frac{2*1}{6*5}=\frac{1}{15}$$

For each orthologous ramp sequence, the expected number of ramp sequences was calculated by multiplying the conditional probability by the total number of ramp sequences with that same taxonomic distribution (e.g., if the dataset contained 15 orthologous genes with ramp sequences where there were three species in the smaller group and seven total species, then the expected number of orthologous ramps across that distribution would be $P*15= \frac{1}{15}*15=1$). A chi square analysis was performed using the expected number of orthologous ramp sequences versus the observed numbers in order to calculate a p-value for the dataset.

### Control comparisons with shortened sequences

We performed an additional control analysis to ensure that ExtRamp identified ramp sequences that likely affected translational efficiency instead of genomic artifacts by removing the first 50 codons in all genes and rerunning our analysis pipeline. Since the ramp sequence generally occurs within the first 50 codons of a gene, we expected this control analysis to identify significantly fewer ramp sequences than the original dataset. We assessed this difference using a chi square statistic and p-value.

### Recovering phylogenies using aligned sequence data

In order to investigate the hypothesis that nucleotides in ramp sequences provide a different phylogenetic signal than other portions of the gene, the aligned sequences were analyzed using maximum likelihood and parsimony. Ramp sequences for each orthologous group were aligned using Clustal Omega^35^ (see Supplementary Note S5 for the command). Sequences were aligned using nucleotide sequence alignment as opposed to amino acid sequence alignment to accommodate potential differences in splice site reading frames between species. Nucleotide sequence alignments allow homologous genes to be aligned that may contain dual-coding exons, which occur when one portion of a sequence can be encoded using different reading frames.

The character matrix was encoded by first concatenating the aligned ramp sequences from each ortholog. Then, if an ortholog was not present in a species, each nucleotide character for that sequence was encoded as a '?' for missing data. The max was then used in IQ-TREE^28^ to select the best model^36^ and perform a maximum likelihood estimation of the phylogeny. The matrix was also used in TNT to recover phylogenies using parsimony.

Phylogenies were similarly recovered using the aligned sequence after the ramp and the complete gene sequence for each orthologous gene. For the maximum likelihood analysis, the size of the dataset for the portion after the ramp sequence and the complete sequence rendered the automatic model selection impractical due to computational demands. Therefore, we selected the same models that were used on the ramp sequence to evaluate the gene sequence after the ramp sequence and the complete gene sequence, which were GTR + F + R5 for Mammalia and GTR + F + R8 for non-mammalian vertebrates.

## Results

### Data analyzed

The original NCBI dataset consisted of a latest assembly version from each species in Mammalia and non-mammalian vertebrates. Ramp sequences were then extracted from all species. In order to limit the effects of missing data, only orthologous genes that contained a ramp in at least 5% of the available species were included in the analysis. Additionally, species were removed if they contained less than 5% of the included orthologous genes. Table 1 shows the resulting dataset that was included in the phylogenetic algorithms.

**Table 1:** The number of species and orthologs for each of the taxonomic groups are reported for the number of reference genomes contained in the NCBI database, the number that contained ramp sequences, and the number that passed the 5% filter criteria.

Number of species and orthologs analyzed						
Taxonomic group	Species	Species containing ramp sequences	Species after filter	Orthologs	Orthologs containing ramp sequences	Orthologs after filter
Mammalia	114	112	110	34,202	16,022	11,670
Non-mammalian vertebrates	133	132	101	41,337	18,779	8,450
Total	247	244	211	75,539	34,801	20,120

### Presence and absence of ramps phylogenies

We recovered phylogenies using a binary matrix that included ramps presence or absence for species and orthologs that passed all filters. Species that did not contain an ortholog were coded as missing. Parsimony analyses were performed using TNT, and all maximum parsimony trees were retained, resulting in two maximum parsimony trees for Mammalia, and two maximum parsimony trees for non-mammalian vertebrates (see Supplementary Figs. S1–S4). Each maximum parsimony tree was compared against the OTL taxonomy using the branch percent identity, and the resulting percentages were averaged for all maximum parsimony trees (see Table 2). Additionally, phylogenies were recovered using maximum likelihood (see Supplementary Figs. S5,S6). Table 2 shows the branch percent identity and compares that percent identity to other previously reported tree reconstruction algorithms and genomic features^24^ including Codon Aversion Motifs, Amino Acid Motifs, Codon Pairing, Feature Frequency Profiles, the word-based methods of CVTree, ACS, Andi, and Filter-spaced word matches, as well as maximum likelihood^24,28,30,37–41^ . A brief description of these algorithms is provided in Supplementary Table S1.

**Table 2:** Branch percent identities of phylogenies recovered using the presence/absence of ramp sequences or other genomic features compared to the OTL.

Branch percent identity compared to the OTL		
	Mammalia	Non-mammalian vertebrates
Ramps—parsimony	70	64
Ramps—maximum likelihood	73	74
Codon aversion motifs	77	66
Amino acid motifs	63	56
Codon pairing	90	77
Feature frequency profiles	52	54
CVTree	69	68
ACS	90	76
Andi	95	81
Filter-space word matches	94	80
Maximum likelihood	93	81

The presence and absence of ramp sequences recovered 70–73% of relationships in the OTL for Mammalia, and 64–74% of relationships in the OTL for non-mammalian vertebrates. Ramp sequences perform comparably to the algorithms based on other genomic features reported.

### Retention index of ramp sequences

**Figure 2:**
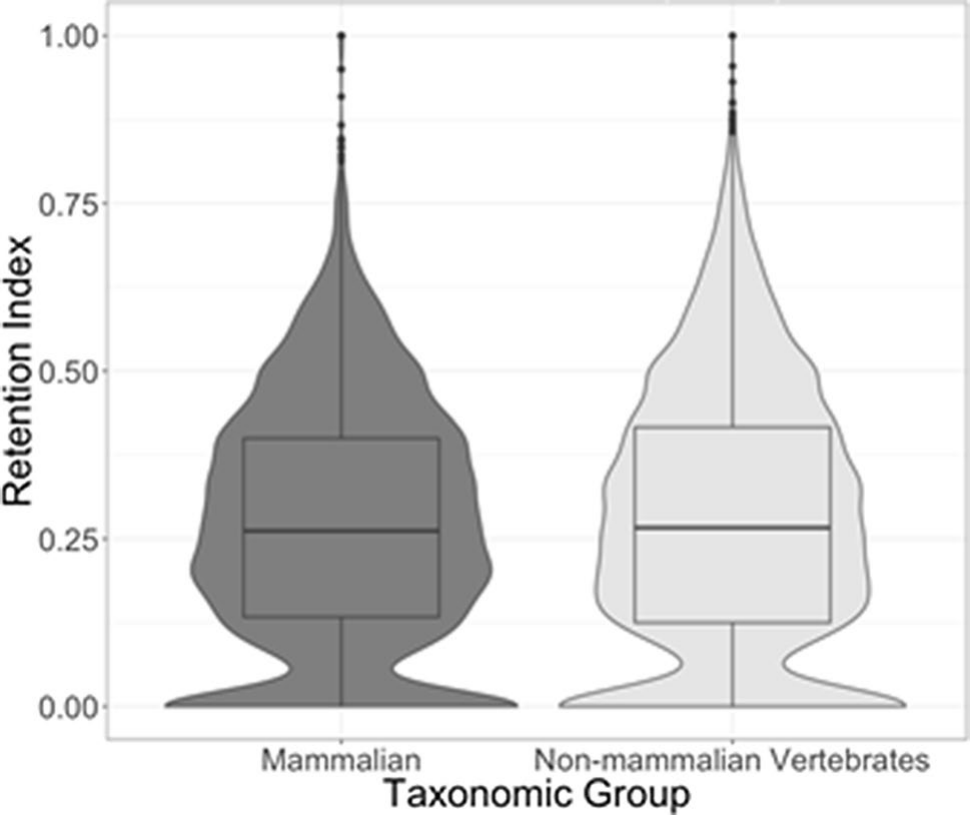
Retention Indices of Ramp Sequences. The distribution of retention indices of each ramp sequence in non-mammalian vertebrates and Mammalia.

The number of parallelisms and reversals for each ramp sequence was counted based on the OTL topology. This number was then used to calculate the retention index of each ramp sequence (Fig. 2).

### Statistical calculations using random permutations

**Figure 3:**
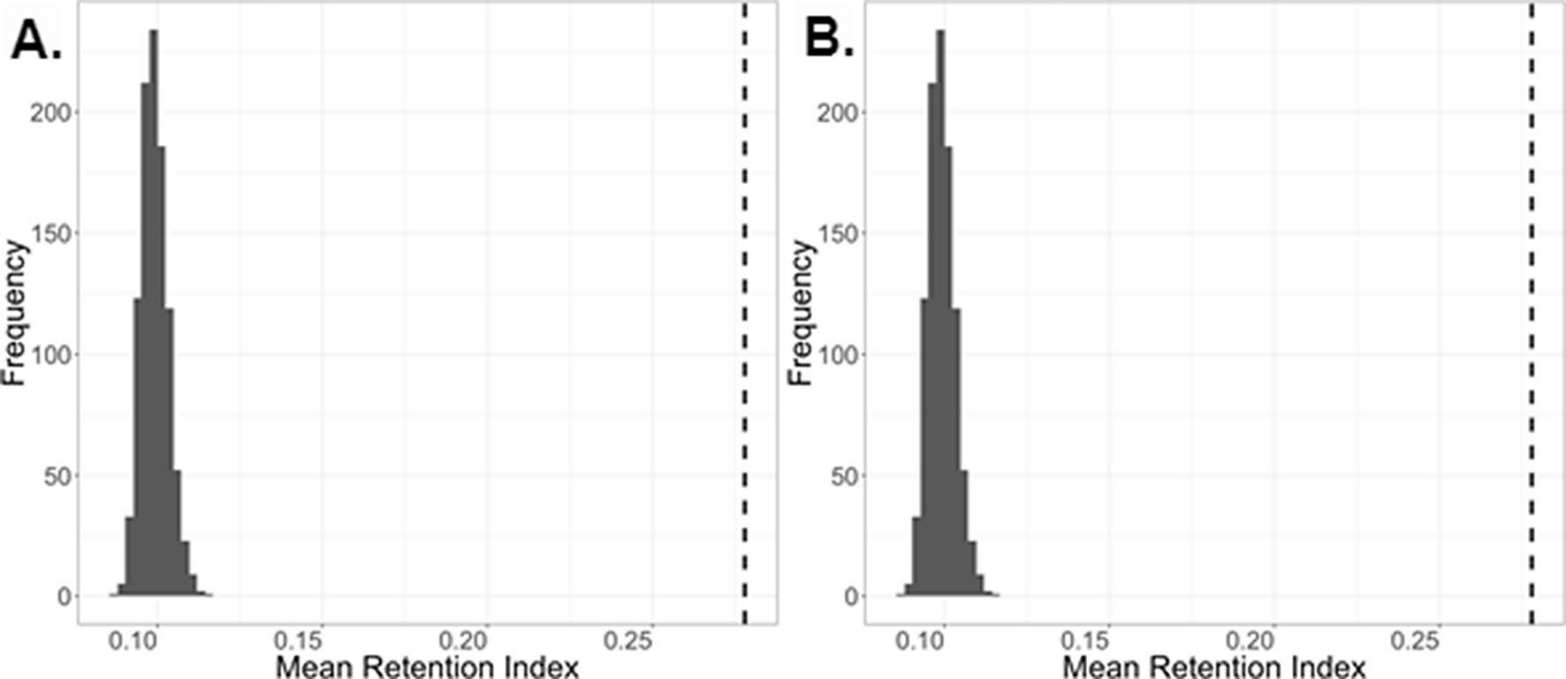
(**A**) The mean retention index for 1000 random permutations in Mammalia, and (**B**) The mean retention index for 1000 random permutations in non-mammalian vertebrates. The actual mean retention index is represented with the dashed line.

We performed 1000 random permutations were performed by shuffling the species in the Open Tree of Life to determine how the retention index of ramp sequences compared to random chance. The observed average retention index for Mammalia was higher than all the random permutations, for an empirical p-value < 0.001. This same result was observed for non-mammalian vertebrates (Fig. 3). Additionally, 81% of vertebrate mammalian ramps and 81.2% of other vertebrate ramps had less parallelisms and reversals than the mean from 1000 randomly permuted trees.

### Orthologous ramp sequences and statistical probabilities

Orthologous ramp sequences were defined as all ramp sequences that had only one origin/loss event on the OTL. The statistical probability that each ramp sequence would be completely homologous by random chance was also calculated. There were 14 completely homologous ramp sequences in Mammalia, and two completely homologous ramp sequences in non-mammalian vertebrates. The statistical probability that each ramp sequence would be completely homologous by random chance was also calculated, and the Bonferroni correction for multiple tests was applied at the 0.05 significance level. Using this threshold, only the kinesin family member 1B (*KIF1B*) gene in non-mammalian vertebrates was statistically significant with a p-value of 6.32 × 10^–7^. Although only one individual homologous gene showed statistical significance, the overall number of completely orthologous ramp sequences was significantly higher than would be expected due to random chance. The chi-square analysis of the observed number versus expected number of orthologous ramp sequences resulted in a p-value < 0.001 for both Mammalia and non-mammalian vertebrates.

### Control comparisons with shortened sequences

A control analysis was performed in which the first 50 codons of all genes were removed in order to assess the validity of targeting ramp sequences that occurred in statistical excess using ExtRamp. In the original gene sequences in Mammalia, ExtRamp identified 378,548 total ramp sequences. In gene sequences that were missing the first 50 codons, only 200,202 ramp sequences were identified. In non-mammalian vertebrates, 169,652 gene sequences were identified with ramp sequences, whereas the shortened sequences yielded only 129,539 gene sequences with ramp sequences. A chi-square test was performed of genes with and without ramps in normal Mammalian genes and shortened Mammalian genes. This resulted in a p-value < 0.000001. Similarly, the chi-square test for non-mammalian vertebrates showed significant results (p-value < 0.000001).

### Aligned sequence phylogenies

The filtered ramp sequences in orthologs were aligned and concatenated to make a matrix of nucleotide character data. This process was done for the extracted ramp sequences, the portion after the ramp sequence, and the combined portions of the genes. Each of these matrices was used to recover phylogenetic trees using parsimony and maximum likelihood. These trees were then compared to the OTL taxonomy using the branch percent identities (Supplementary Fig. S7). Aligned ramp sequences showed lower congruence with the OTL than the portion after the ramp or the complete gene. However, the differences were not statistically significant, indicating that there was no difference in the phylogenetic signal in the nucleotides within the ramp sequence portion of the gene versus the rest of the gene sequence.

## Discussion

Since the first algorithm to extract ramp sequences from single gene sequences was only recently developed, the phylogenetic implications of ramp sequences have previously been unknown. Our comprehensive analyses of ramp sequences in vertebrates suggest that the presence or absence of a ramp sequence in some orthologs is largely congruent with the Open Tree of Life. However, some ramp sequences do not appear to be bound by the same evolutionary constraints. Therefore, while ramp sequences add additional support to a phylogeny across all orthologs, they should be used in conjunction with other phylogenomic methods and may not be suitable for limited analyses of certain genic regions.

A parsimony analysis was able to recover a phylogeny that was relatively similar, as compared to other genomic approaches, to the OTL taxonomy for both Mammalia (70% to 73%) and non-mammalian vertebrates (64% to 74%) by considering only the presence or absence of a ramp sequence in orthologs. These results are comparable to results using other genomic features such as Codon Aversion Motifs, Amino Acid Motifs, Feature Frequency Profiles, and CVTree. The results are slightly lower than Codon Pairing, ACS, Andi, Filter-spaced word matches, and maximum likelihood. These results indicate that considering the presence or absence of a ramp sequence as a morphological genomic character is comparable to other genomic features and may provide additional insights when used in conjunction with these other algorithms.

The retention index analysis of parallelisms and reversals of ramp sequences suggests that ramp sequences contain phylogenetic information. According to the random permutations statistical test, the retention index is higher than would be expected due to random chance (p-value < 0.001) for both Mammalia and non-mammalian vertebrates, with 81% of vertebrate mammalian ramps and 81.2% of other vertebrate ramps having less parallelisms and reversals than the mean from 1000 randomly permuted trees. Additionally, both Mammalia and non-mammalian vertebrates contained more completely orthologous ramp sequences as compared to the OTL than would be expected by random chance (p-value < 0.001). This analysis shows that although about 20% of ramp sequences are more homoplasious than random chance, the overall usage of ramp sequences shows statistically significant levels of homology as compared to the OTL topology. Therefore, ramp sequence phylogenetic signal across all orthologs indicates strong similarity to the OTL.

Separate analyses were performed of aligned ramp sequences, the gene sequence after the ramp sequence, and the complete gene sequence so that the phylogenetic signal of different orthologous regions of could be compared. These analyses were completed using both maximum likelihood and parsimony, and the branch percent comparisons were compared for the OTL taxonomy. The results indicated that ramp sequences show less congruence to the OTL than the rest of the sequence portion. However, this result was not statistically significant and may have been confounded by the small length of the ramp sequence relative to the rest of the gene. Additionally, this analysis supports our binary encoding of ramp sequences to elucidate the most congruent phylogenetic signal compared to the OTL.

These analyses collectively show that ramp sequence usage tracks speciation more frequently than random chance in Mammalia and non-mammalian vertebrates. Additionally, it is likely that ramp sequences will provide additional phylogenetic information for other taxonomic groups as more orthologs are identified and annotated across more diverse species. Phylogenetic analyses using a single ortholog or a subset of orthologs might not benefit from incorporating ramp sequences because a small, but significant, portion of ramp sequences do not track the OTL. However, phylogenomic analyses that use various orthologous sequences should use ramp sequences because the overall phylogenetic signal of ramp sequences strongly supports established phylogenies and is comparable to other genomic features.

## Supplementary Information

Supplementary Information.

## Data Availability

The datasets analyzed during the current study were downloaded from the National Center for Biotechnology Information (NCBI) database^19,42^ in August 2018 using their FTP site: ftp://ftp.ncbi.nlm.nih.gov/genomes/refseq/. The data, scripts and programs used in this manuscript are freely available at https://github.com/ridgelab/Ramps_Phylogeny.
