# Supplementary material for: A comprehensive analysis of the phylogenetic signal in ramp sequences in 211 vertebrates
Source: Sci Rep. 2021 Jan 12;11:622. doi: 10.1038/s41598-020-78803-3 (PMC7803996; doi:10.1038/s41598-020-78803-3)
Supplement: Supplementary file 1 — Supplementary Information. [file 41598_2020_78803_MOESM1_ESM.docx]

Supplementary Information for A Comprehensive Analysis of the Phylogenetic Signal in Ramp Sequences in 211 Vertebrates

Lauren M. McKinnon, Justin B. Miller, Michael F. Whiting, John S.K. Kauwe, and Perry G. Ridge

**Table of Contents**

[SUPPLEMENTARY NOTES 2](#_Toc52808735)

[Note S1: Vertebrate Mammalian Species Analyzed: 2](#_Toc52808736)

[Note S2: Other Vertebrate Species Analyzed: 2](#_Toc52808737)

[Note S3: ExtRamp Command: 3](#_Toc52808738)

[Note S4: OTL Command: 3](#_Toc52808739)

[Note S5: Clustal Omega Command: 3](#_Toc52808740)

[SUPPLEMENTARY FIGURES 4](#_Toc52808741)

[Fig. S1: Mammals parsimony tree #1 4](#_Toc52808742)

[Fig. S2: Mammals parsimony tree #2 5](#_Toc52808743)

[Fig. S3: Mammals maximum likelihood tree 6](#_Toc52808744)

[Fig. S4: Non-mammalian vertebrates parsimony tree #1 7](#_Toc52808745)

[Fig. S5: Non-mammalian vertebrates parsimony tree #2 8](#_Toc52808746)

[Fig. S6: Non-mammalian vertebrates maximum likelihood tree 9](#_Toc52808747)

[Fig. S7: Comparisons of Ramp Phylogenies with the OTL 10](#_Toc52808748)

[SUPPLEMENTARY TABLES 11](#_Toc52808749)

[Table S1: A brief description of phylogenetic algorithms 11](#_Toc52808750)

# SUPPLEMENTARY NOTES

## Note S1: Vertebrate Mammalian Species Analyzed:

The following 114 vertebrate mammalian species were included in this analysis: *Acinonyx jubatus, Ailuropoda melanoleuca, Aotus nancymaae, Balaenoptera acutorostrata, Bison bison, Bos indicus, Bos mutus, Bos taurus, Bubalus bubalis, Callithrix jacchus, Camelus bactrianus, Camelus dromedarius, Camelus ferus, Canis lupus, Capra hircus, Carlito syrichta, Castor canadensis, Cavia porcellus, Cebus capucinus, Ceratotherium simum, Cercocebus atys, Chinchilla lanigera, Chlorocebus sabaeus, Chrysochloris asiatica, Colobus angolensis, Condylura cristata, Cricetulus griseus, Dasypus novemcinctus, Delphinapterus leucas, Desmodus rotundus, Dipodomys ordii, Echinops telfairi, Elephantulus edwardii, Enhydra lutris, Eptesicus fuscus, Equus asinus, Equus caballus, Equus przewalskii, Erinaceus europaeus, Felis catus, Fukomys damarensis, Galeopterus variegatus, Gorilla gorilla, Heterocephalus glaber, Hipposideros armiger, Homo sapiens, Ictidomys tridecemlineatus, Jaculus jaculus, Leptonychotes weddellii, Lipotes vexillifer, Loxodonta africana, Macaca fascicularis, Macaca mulatta, Macaca nemestrina, Mandrillus leucophaeus, Manis javanica, Marmota marmota, Meriones unguiculatus, Mesocricetus auratus, Microcebus murinus, Microtus ochrogaster, Miniopterus natalensis, Monodelphis domestica, Mus caroli, Mus musculus, Mus pahari, Mustela putorius, Myotis brandtii, Myotis davidii, Myotis lucifugus, Nannospalax galili, Neomonachus schauinslandi, Neophocaena asiaeorientalis, Nomascus leucogenys, Ochotona princeps, Octodon degus, Odobenus rosmarus, Odocoileus virginianus, Orcinus orca, Ornithorhynchus anatinus, Orycteropus afer, Oryctolagus cuniculus, Otolemur garnettii, Ovis aries, Pan paniscus, Panthera pardus, Panthera tigris, Pantholops hodgsonii, Pan troglodytes, Papio anubis, Peromyscus maniculatus, Phascolarctos cinereus, Physeter catodon, Piliocolobus tephrosceles, Pongo abelii, Propithecus coquereli, Pteropus alecto, Pteropus vampyrus, Puma concolor, Rattus norvegicus, Rhinolophus sinicus, Rhinopithecus bieti, Rhinopithecus roxellana, Rousettus aegyptiacus, Saimiri boliviensis, Sarcophilus harrisii, Sorex araneus, Sus scrofa, Theropithecus gelada, Trichechus manatus, Tupaia chinensis, Tursiops truncatus, Ursus maritimus,* and *Vicugna pacos.*

## Note S2: Other Vertebrate Species Analyzed:

The following 133 other vertebrate species were analyzed: *Acanthisitta chloris, Acanthochromis polyacanthus, Alligator mississippiensis, Alligator sinensis, Amphiprion ocellaris, Anas platyrhynchos, Anolis carolinensis, Anser cygnoides, Antrostomus carolinensis, Apaloderma vittatum, Aptenodytes forsteri, Apteryx australis, Aquila chrysaetos, Astyanax mexicanus, Austrofundulus limnaeus, Balearica regulorum, Boleophthalmus pectinirostris, Buceros rhinoceros, Calidris pugnax, Callorhinchus milii, Calypte anna, Cariama cristata, Chaetura pelagica, Charadrius vociferus, Chelonia mydas, Chlamydotis macqueenii, Chrysemys picta, Clupea harengus, Colius striatus, Columba livia, Corvus brachyrhynchos, Corvus cornix, Coturnix japonica, Crocodylus porosus, Cuculus canorus, Cyanistes caeruleus, Cynoglossus semilaevis, Cyprinodon variegatus, Cyprinus carpio, Danio rerio, Dromaius novaehollandiae, Egretta garzetta, Esox lucius, Eurypyga helias, Falco cherrug, Falco peregrinus, Ficedula albicollis, Fulmarus glacialis, Fundulus heteroclitus, Gallus gallus, Gavialis gangeticus, Gavia stellata, Gekko japonicus, Geospiza fortis, Haliaeetus albicilla, Haliaeetus leucocephalus, Haplochromis burtoni, Hippocampus comes, Ictalurus punctatus, Kryptolebias marmoratus, Labrus bergylta, Larimichthys crocea, Lates calcarifer, Latimeria chalumnae, Lepidothrix coronata, Lepisosteus oculatus, Leptosomus discolor, Lonchura striata, Maylandia zebra, Meleagris gallopavo, Melopsittacus undulatus, Merops nubicus, Mesitornis unicolor, Monopterus albus, Nanorana parkeri, Neolamprologus brichardi, Nestor notabilis, Nipponia nippon, Nothobranchius furzeri, Nothoprocta perdicaria, Notothenia coriiceps, Numida meleagris, Oncorhynchus kisutch, Oncorhynchus mykiss, Oncorhynchus tshawytscha, Opisthocomus hoazin, Oreochromis niloticus, Oryzias latipes, Oryzias melastigma, Paralichthys olivaceus, Paramormyrops kingsleyae, Parus major, Pelecanus crispus, Pelodiscus sinensis, Phaethon lepturus, Phalacrocorax carbo, Picoides pubescens, Poecilia formosa, Poecilia latipinna, Poecilia mexicana, Poecilia reticulata, Pogona vitticeps, Protobothrops mucrosquamatus, Pseudopodoces humilis, Pterocles gutturalis, Pundamilia nyererei, Pygocentrus nattereri, Pygoscelis adeliae, Python bivittatus, Rhincodon typus, Salmo salar, Salvelinus alpinus, Scleropages formosus, Serinus canaria, Seriola dumerili, Seriola lalandi, Sinocyclocheilus anshuiensis, Sinocyclocheilus grahami, Sinocyclocheilus rhinocerous, Stegastes partitus, Struthio camelus, Sturnus vulgaris, Taeniopygia guttata, Takifugu rubripes, Tauraco erythrolophus, Terrapene mexicana, Thamnophis sirtalis, Tinamus guttatus, Tyto alba, Xenopus laevis, Xenopus tropicalis, Xiphophorus maculatus,* and *Zonotrichia albicollis.*

## Note S3: ExtRamp Command:

The following command generated a FASTA file of ramp sequences ${ramps_output} and a FASTA file of the sequence after the ramp ${after_output}.

python3 ExtRamp.py -i ${ref_fasta} -o ${ramps_output} -x ${after_output}

## Note S4: OTL Command:

Reference phylogenies were retreived from the OTL database using the getOTL.py as found in the README file (<https://github.com/ridgelab/cam>), where ${input} is a list of species, and ${output} is the output file containing the phylogeny.

python getOTLtree.py -i ${input} -o ${output}

## Note S5: Clustal Omega Command:

Sequences were aligned using Clustal Omega. The following command uses a FASTA file ${input} of sequences and generates a FASTA file ${output} of aligned sequences.

clustalo -i ${input} > ${output}

# SUPPLEMENTARY FIGURES

## Fig. S1: Mammals parsimony tree #1


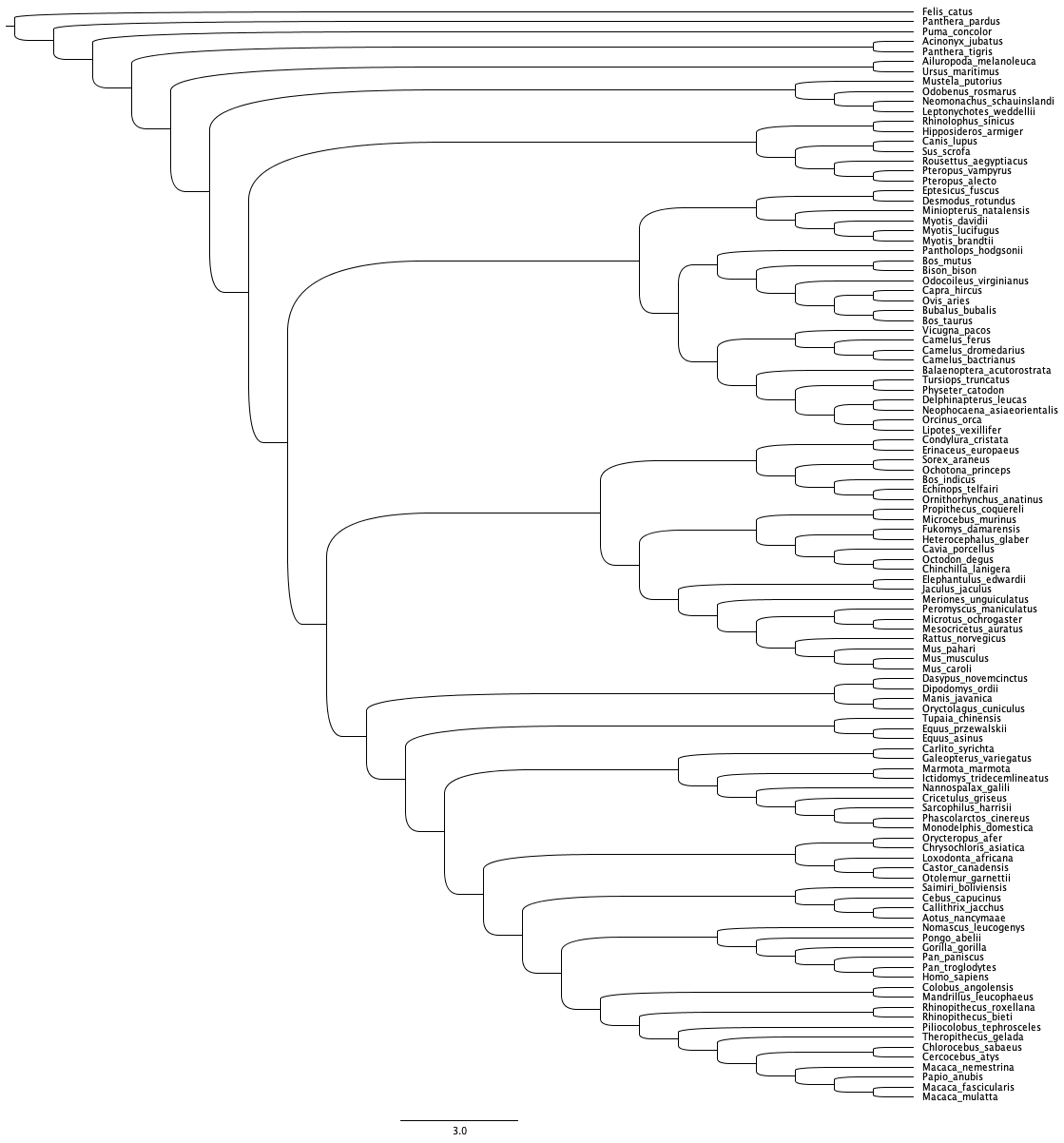


## Fig. S2: Mammals parsimony tree #2


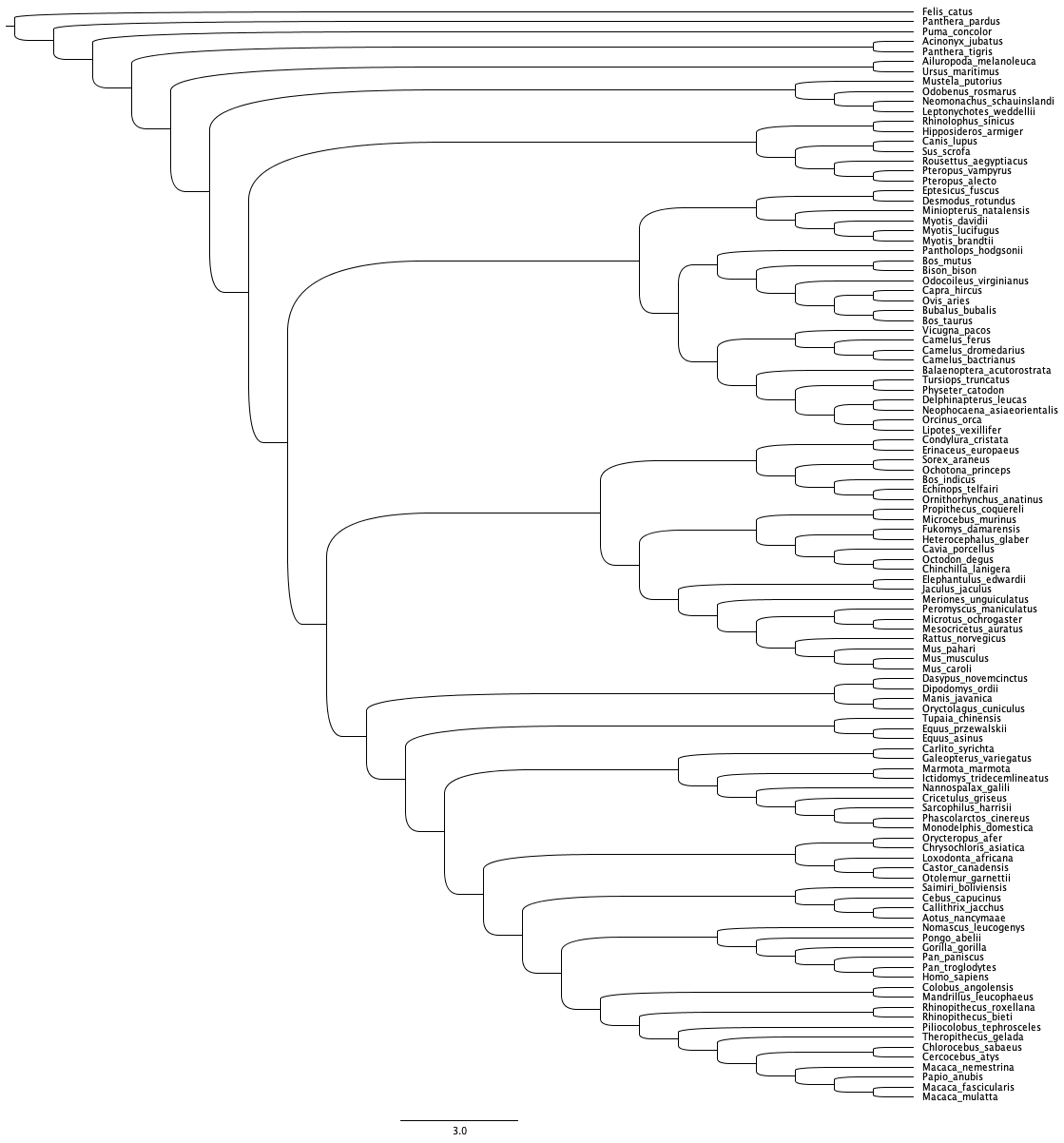


## Fig. S3: Mammals maximum likelihood tree


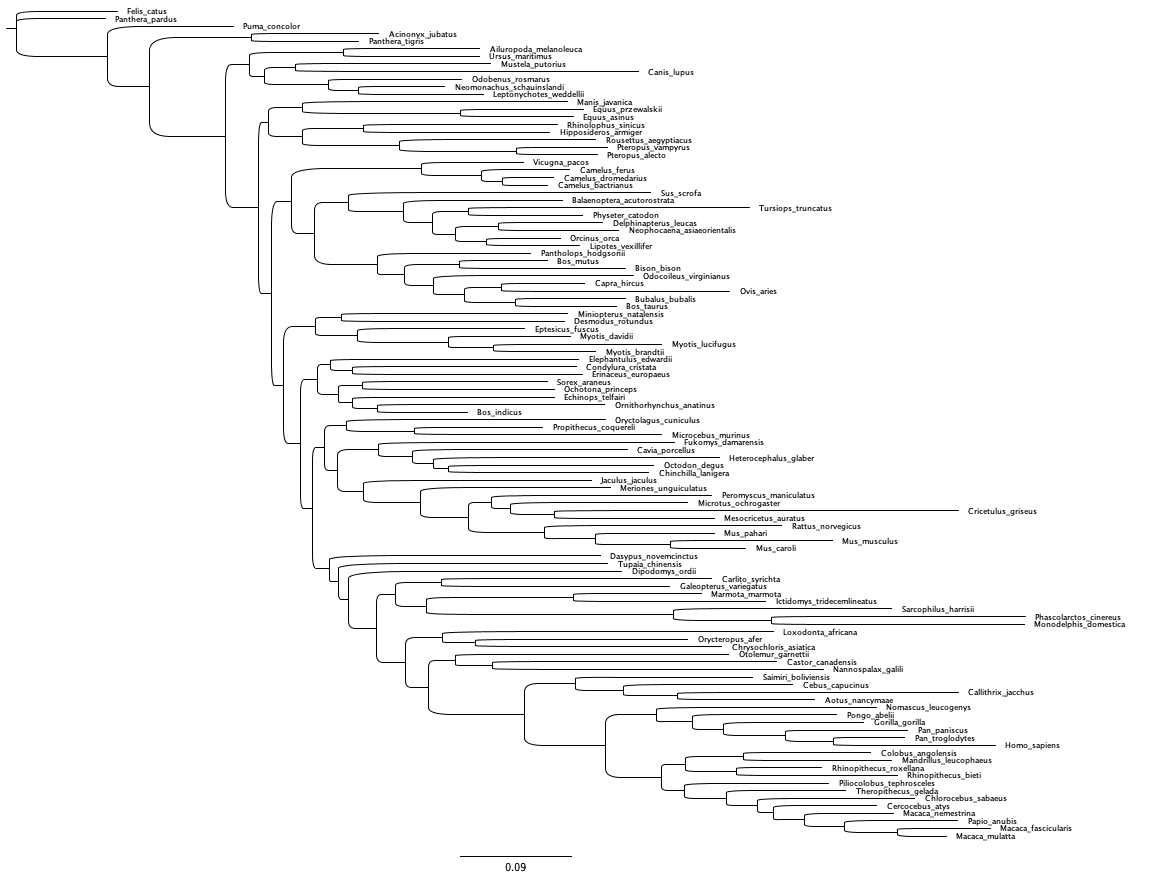


Fig. S4: Non-mammalian vertebrates parsimony tree #1
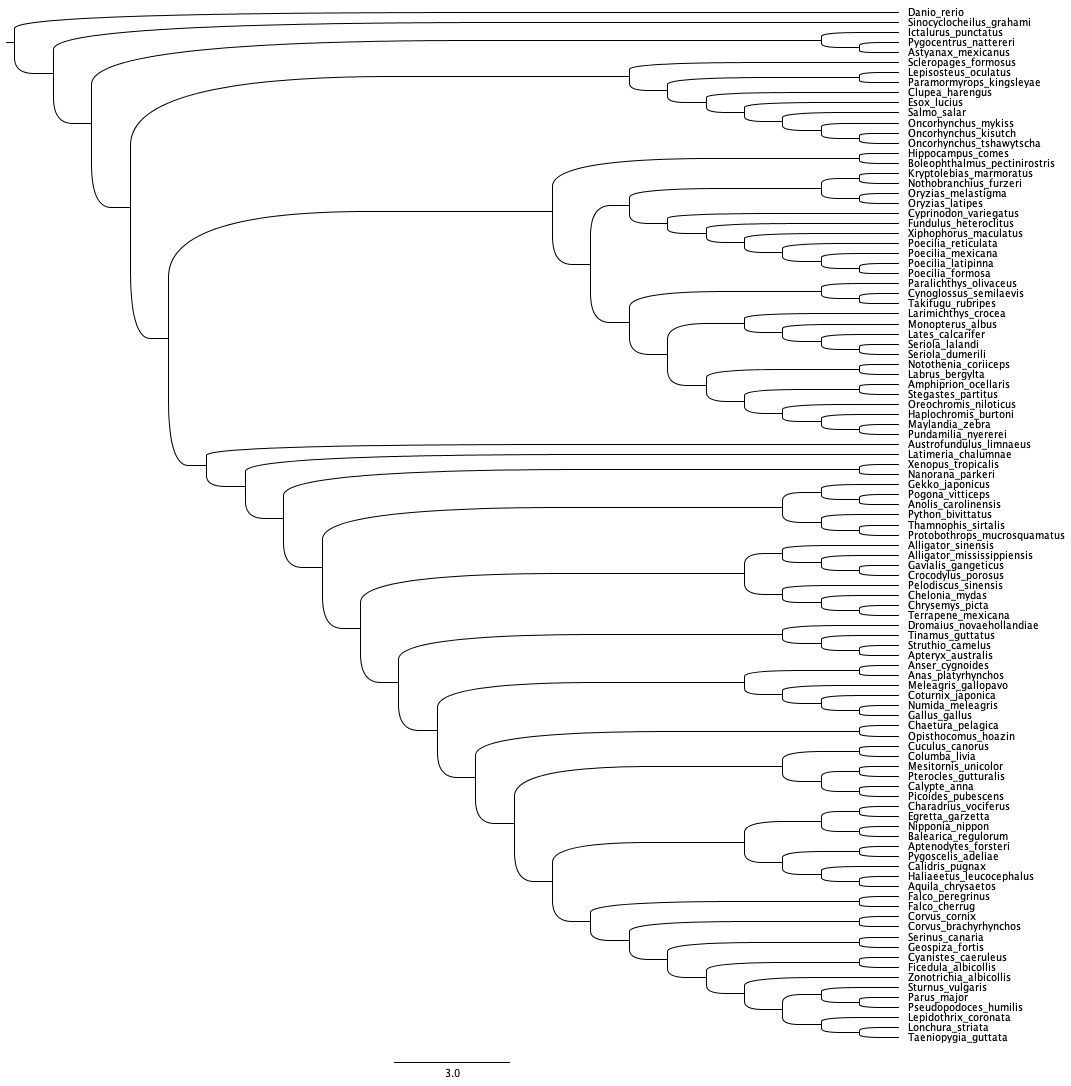


## Fig. S5: Non-mammalian vertebrates parsimony tree #2


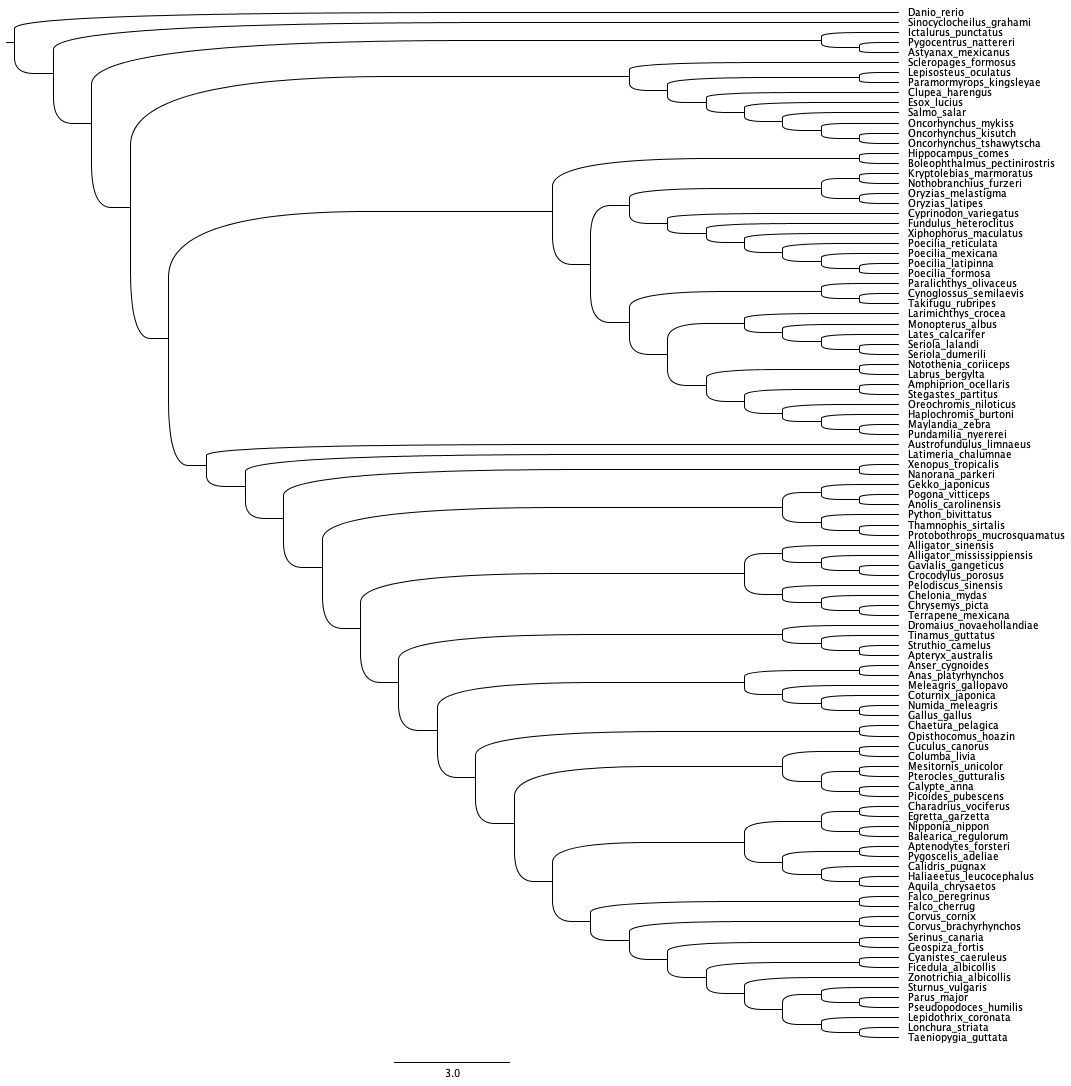


## Fig. S6: Non-mammalian vertebrates maximum likelihood tree


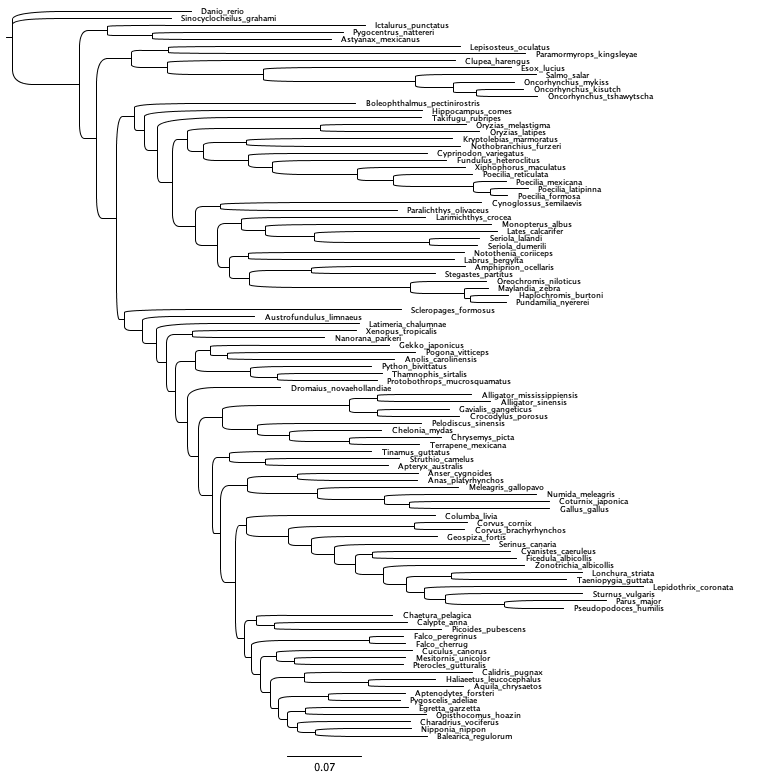


## Fig. S7: Comparisons of Ramp Phylogenies with the OTL


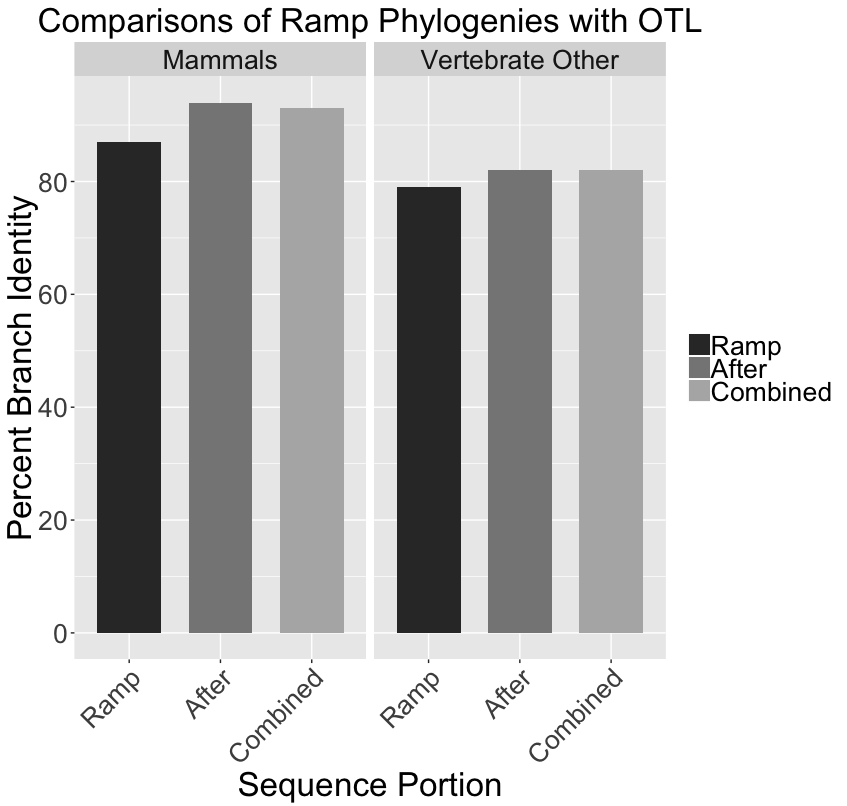


Phylogenies recovered using ramp sequences, the portion after the ramp sequence, and full sequences were compared to the OTL taxonomy using branch percent identity.

# SUPPLEMENTARY TABLES

## Table S1: A brief description of phylogenetic algorithms

| **Algorithm** | **Description** |
| --- | --- |
| Codon Aversion Motifs | Motifs of codons that are completely avoided are constructed for each gene. The distance between two species is calculated as the intersection of codon aversion motifs divided by the total number of unique motifs in the species with less motifs. |
| Amino Acid Motifs | Distance is calculated in the same manner as in Codon Aversion Motifs using amino acids instead of codons. |
| Codon Pairing | Codon pairing occurs when two codons that encode the same amino acid are located within a ribosomal window. Codon pairing was analyzed in a parsimony framework to infer a phylogeny. |
| Feature Frequency Profiles | The frequency of different k-mers is calculated, and the resulting profiles are compared between species to calculate a distance. |
| CVTree | Frequencies of words of a given length are calculated using composition vectors and then normalized based on the expected frequencies predicted by random chance. These frequencies are used to calculate a distance between species. |
| ACS | At each index of a gene, the longest matching substring is found in the second sequence. The average of these matching substrings is used to calculate a distance. |
| Andi | Andi creates micro-alignments between two sequences. It searches for mismatches that are bracketed by long, exact matches. These mismatches are then combined into a single matrix to estimate a mutation rate. |
| Filter-spaced word matches | Filter-spaced word matches finds matching-spaced words between sequences in a similar manner to Andi. It then adjusts the mutation rate, accounting for pattern matches caused by random chance. |
| Maximum Likelihood | This common alignment-based technique determines species relationships by finding the most likely phylogenetic tree by incorporating a model of evolution that includes estimated parameters such as transition/transversion frequencies, nucleotide frequencies, etc. |
